# Concomitant colour polymorphs of (*Z*)-*N*-(4-fluoro­phen­yl)-2-oxo­propane­hydrazonoyl chloride

**DOI:** 10.1107/S2053229625006618

**Published:** 2025-07-30

**Authors:** Lisa Müller, Richard Goddard, Petra Frohberg, Rüdiger W. Seidel

**Affiliations:** a Institut für Pharmazie, Martin-Luther-Universität Halle-Wittenberg, Wolfgang-Langenbeck-Strasse 4, 06120 Halle (Saale), Germany; b Max-Planck-Institut für Kohlenforschung, Kaiser-Wilhelm-Platz 1, 45470 Mülheim an der Ruhr, Germany; The University of Western Australia, Australia

**Keywords:** hydrazonoyl chloride, hy­dro­gen bonding, colour polymorphism, Hirshfeld atom refinement, crystal structure

## Abstract

The title com­pound, (*Z*)-*N*-(fluoro­phen­yl)-2-oxo­propane­hydrazonoyl chloride, C_9_H_8_ClFN_2_O, was found to form concomitant colour polymorphs upon recrystallization from acetone. Block-shaped pale-orange crystals of form I (space group *P*2_1_/*n*, No. 14) and prism-shaped yellow crystals of form II (space group *P*2_1_/*c*, No. 14) both belong to the monoclinic crystal system. N—H⋯O hy­dro­gen bonds resulting in zigzag chains [graph-set descriptor *C*(6)] are the dominating inter­molecular inter­actions in both crystal forms. The hy­dro­gen-bonded zigzag chains so formed extend by 2_1_ screw symmetry in form I and by *c*-glide symmetry in form II, and are arranged in layers, which are more corrugated in form II than in form I. The polymorphs are virtually indistinguishable by their calculated densities, packing indices and melting points. Form I sublimes to yield crystals of form II, whereas form II sublimes to afford crystals of the same polymorph with characteristic crystal morphology.

## Abstract 2

(*Z*)-*N*-(4-Fluoro­phen­yl)-2-oxo­propane­hydrazonoyl chloride forms concomitant colour polymorphs, namely, block-shaped pale-orange crystals of form I (space group *P*2_1_/*n*, *Z* = 4) and needle-shaped pale-yellow crystals of form II (space group *P*2_1_/*c*, *Z* = 4).

## Introduction

Crystal polymorphism is the phenomenon in which a chemical com­pound can exist in more than one crystal form (Cruz-Cabeza *et al.*, 2020 ▸). Thus, the atoms or mol­ecules of the same substance can arrange into different patterns in the solid state. The different crystal forms, *i.e.* polymorphs, can exhibit different physical properties, such as melting point, solubility, hardness, crystal shape and optical properties, including colour. Colour polymorphism is a relatively rare phenomenon in mol­ecular crystals, with a limited number of examples described in the literature (Nogueira *et al.*, 2020 ▸). A unique example is 5-methyl-2-[(2-nitro­phen­yl)amino]­thio­phene-3-car­bo­nitrile, which has up to 14 known polymorphs, the colours of which vary between red, through orange to yellow, giving rise to the acronym ROY (Weatherston *et al.*, 2025 ▸).
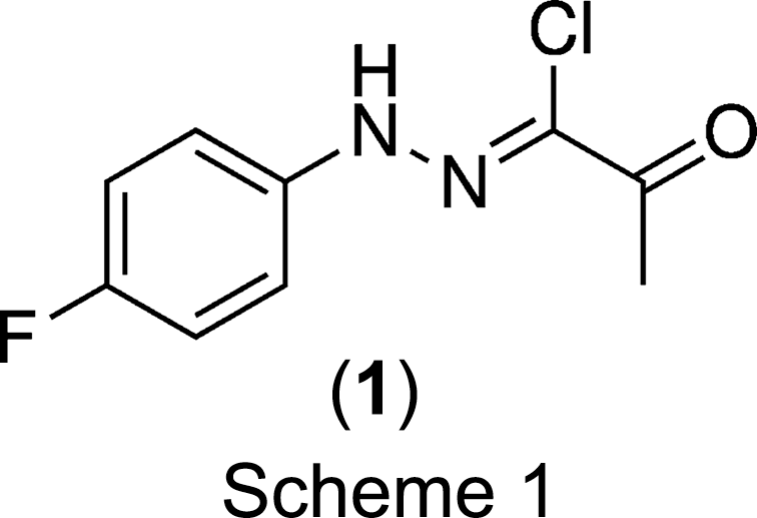


In the course of our studies on heterocyclic com­pounds as lipoxygenase inhibitors (Frohberg *et al.*, 1995 ▸), we serendipitously discovered colour polymorphism of the precursor (*Z*)-*N*-(fluoro­phen­yl)-2-oxo­propane­hydrazonoyl chloride, (**1**) (Scheme 1), upon recrystallization from acetone. The com­pound can be con­veni­ent­ly synthesized from 3-chloro­pentane-2,4-dione and 4-fluoro­benzene­diazo­nium chloride (Biere *et al.*, 1982 ▸) by em­ploying the Japp–Klingemann reaction (Japp & Klingemann, 1888 ▸). For the synthesis and biological activity of hydrazonoyl halides, we direct the inter­ested reader to the review by Sayed *et al.* (2020 ▸). We have also investigated the solid-state structures of phenyl­hydrazonoyl chlorides previously (Frohberg *et al.*, 2002 ▸), but, as of now, examples characterized by X-ray crystallography remain scarce.

A search of the Cambridge Structural Database (CSD; Groom *et al.*, 2016 ▸) for *N*-phenyl-2-oxo­propane­hydrazonoyl halides yielded the crystal structures of the unsubstituted phenyl derivative (CSD refcode XEBWIM; Abdel-Aziz *et al.*, 2012 ▸) and the 4-meth­oxy­phenyl (AWUXAS; Asiri *et al.*, 2011*a* ▸), 4-chloro­phenyl (AWUXEW; Asiri *et al.*, 2011*b* ▸) and 4-nitro­phenyl (AWUXIA; Asiri *et al.*, 2011*c* ▸) derivatives, but the title com­pound, (**1**), has not been structurally characterized by X-ray crystallography, as far as we are able to ascertain. In this article, we report the structure elucidation of two colour polymorphs of (**1**), which crystallized concomitantly, *i.e.* simultaneously, from the same solution (Bernstein *et al.*, 1999 ▸).

## Experimental

### General

Starting materials were obtained from commercial sources and were used as received. Solvents were of reagent grade quality. NMR spectra were recorded on an Agilent Technologies 400 MHz VNMRS NMR spectrometer (abbreviations: *s* = singlet, *d* = doublet and *m* = multiplet). Chemical shifts are reported relative to the residual solvent signals of chloro­form-*d* (δ_H_ = 7.26 ppm and δ_C_ = 77.10 ppm). Melting point determinations and sublimation experiments were performed using a Reichert hot stage mounted on a Nikon SMZ 1500 microscope. Differential scanning calorimetry (DSC) was conducted on a Mettler Toledo Thermal Analysis System DSC 5+, using the *STAR^e^* software (Version 19.00). FT–IR spectra were recorded on a Bruker Tensor 27 spectrometer with a diamond atten­uated total reflectance (ATR) unit.

### Synthesis and crystallization

4-Fluoro­aniline (11.1 g, 0.1 mol) was dissolved in 60 ml of hydro­chloric acid (6 mol l^−1^). After cooling to 273–278 K in an ice bath, a solution of sodium nitrite (6.9 g, 0.1 mol) in 20 ml of deionized water was added with stirring. A freshly prepared solution of 4-fluoro­benzene­diazo­nium chloride was added dropwise to a solution of 3-chloro­pentane-2,4-dione (13.5 g, 0.1 mol) and 40 g of anhydrous sodium acetate in 250 ml of methanol. The tem­per­a­ture of the reaction mixture was maintained at 278–283 K with an ice bath. After stirring for 2 h, the precipitate was separated by filtration, washed with deionized water, dried in air and recrystallized twice from acetone to yield (**1**) (yield: 12.02 g, 0.056 mol, 56%). ^1^H NMR (402 MHz, chloro­form-*d*): δ 8.45 (*s*, 1H, NH), 7.21–7.15 (*m*, 2H, phenyl-H), 7.07–7.00 (*m*, 2H, phenyl-H), 2.53 (*s*, 3H, CH_3_) ppm. ^13^C NMR (101 MHz, chloro­form-*d*): δ 188.2, 159.1 (*d*, ^1^*J*_C,F_ = 242 Hz), 137.7 (*d*, ^4^*J*_C,F_ = 3 Hz), 125.2, 116.3 (*d*, ^2^*J*_C,F_ = 23 Hz), 115.8 (*d*, ^3^*J*_C,F_ = 8 Hz), 25.2 ppm.

Pale-orange crystals of form I and and pale-yellow crystals of form II suitable for single-crystal X-ray diffraction analysis were obtained when a saturated solution of (**1**) in acetone in a 4 ml borosilicate glass vial slowly evaporated to dryness after standing at room tem­per­a­ture for several days [see Fig. S1(*a*) in the supporting information]. Subsequently, the two crystal forms were separated manually [Fig. S1(*b*)].

### X-ray crystallography

The crystal structures of forms I and II were both initially refined by independent atom model (IAM) refinement using *SHELXL2019* (Sheldrick, 2015*b* ▸). The final structure refinement was performed with aspherical atomic form factors using *NoSpherA2* (Kleemiss *et al.*, 2021 ▸; Midgley *et al.*, 2021 ▸) in *OLEX2* (Dolomanov *et al.*, 2009 ▸). Hirshfeld-partitioned electron density was calculated with *ORCA* (Version 5.0; Neese *et al.*, 2020 ▸) using the B3LYP hybrid functional (Becke, 1993 ▸; Lee *et al.*, 1988 ▸) and the def2-TZVPP basis set (Weigend & Ahlrichs, 2005 ▸). Positions and isotropic atomic displacement parameters were refined freely for all H atoms. For form I, atom Cl1 was refined anharmonically using a Gram Charlier expansion to fourth order implemented in *OLEX2*, although not strictly obeying Kuhs’ rule (Kuhs, 1988 ▸), according to which an estimated resolution of (sin θ/λ)_max_ = 0.56 Å^−1^ (*cf.* Table 1 ▸) is required to resolve anharmonic atomic displacements. Nonetheless, the anharmonic refinement of Cl1 in form I resulted in a flat difference electron density, as com­pared to Δρ_max_, Δρ_min_ = 0.61, −0.22 e Å^−3^ with harmonic refinement (*cf.* Table 1 ▸) and a decrease in *wR*(*F*^2^) from 0.0804 to 0.0752, despite the additional 25 parameters associated with the anharmonic refinement. Crystal data, data collection and structure refinement details are summarized in Table 1 ▸.

Root-mean-square (r.m.s.) deviations between the mol­ecular structures in forms I and II were calculated with *Mercury* (Macrae *et al.*, 2020 ▸), and r.m.s. deviations of the mol­ecules from exact point-group symmetry, as well as packing indices, were calculated with *PLATON* (Spek, 2020 ▸). Hirshfeld surface analysis was carried out with *CrystalExplorer* (Spackman *et al.*, 2021 ▸).

## Results and discussion

**Figure 1:**
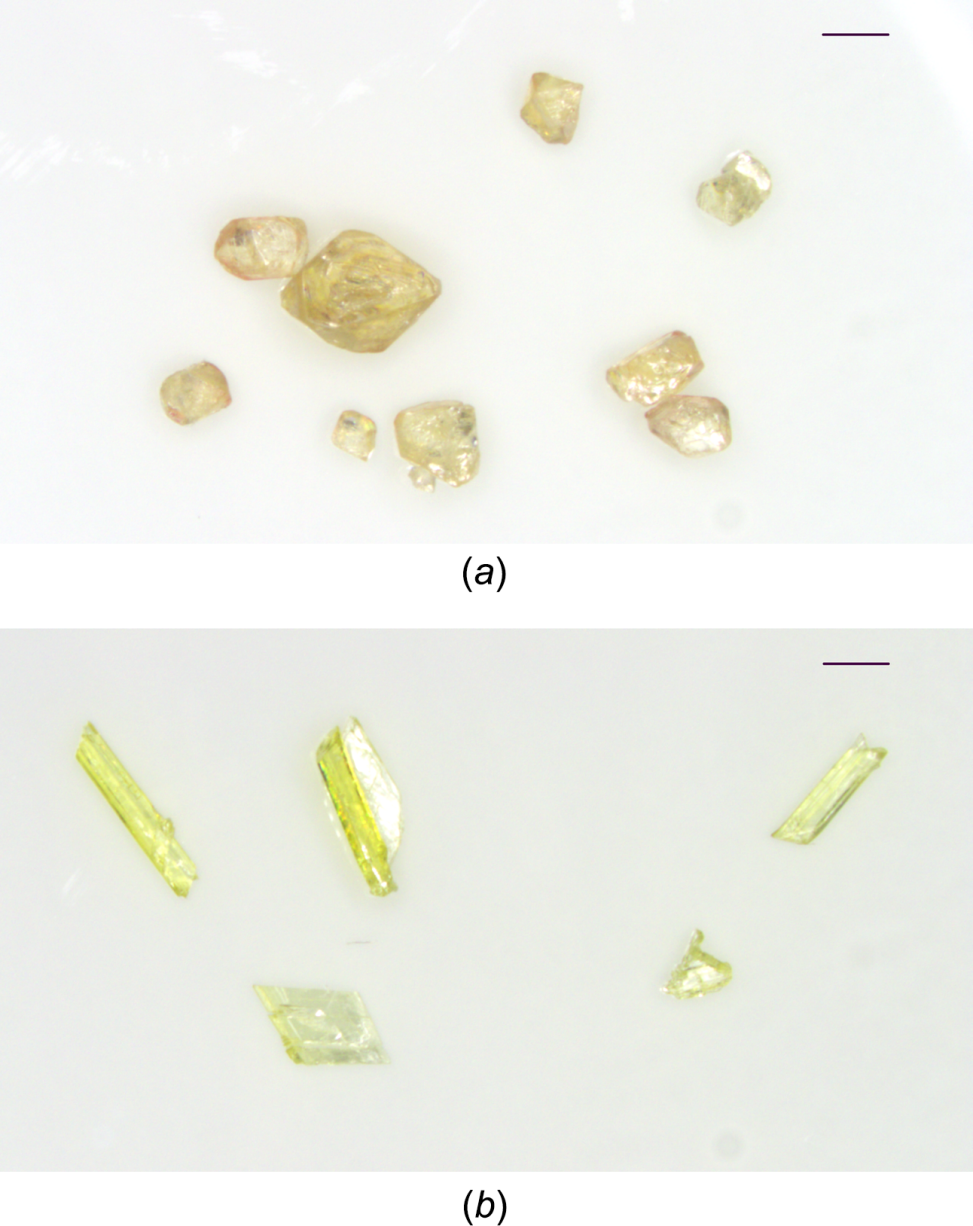
Microscope images of (*a*) crystal form I and (*b*) crystal form II of (**1**). Scale bars = 1 mm.

The title com­pound, (**1**), was found to crystallize concomitantly in two polymorphic forms from acetone. Crystal forms I and II can be readily distinguished from one another by their colours and external shapes. Polymorph I forms block-shaped pale-orange crystals, while polymorph II forms pale-yellow prisms (Fig. 1 ▸). The crystal and mol­ecular structures of both polymorphs were elucidated by single-crystal X-ray diffraction analysis.

**Figure 2:**
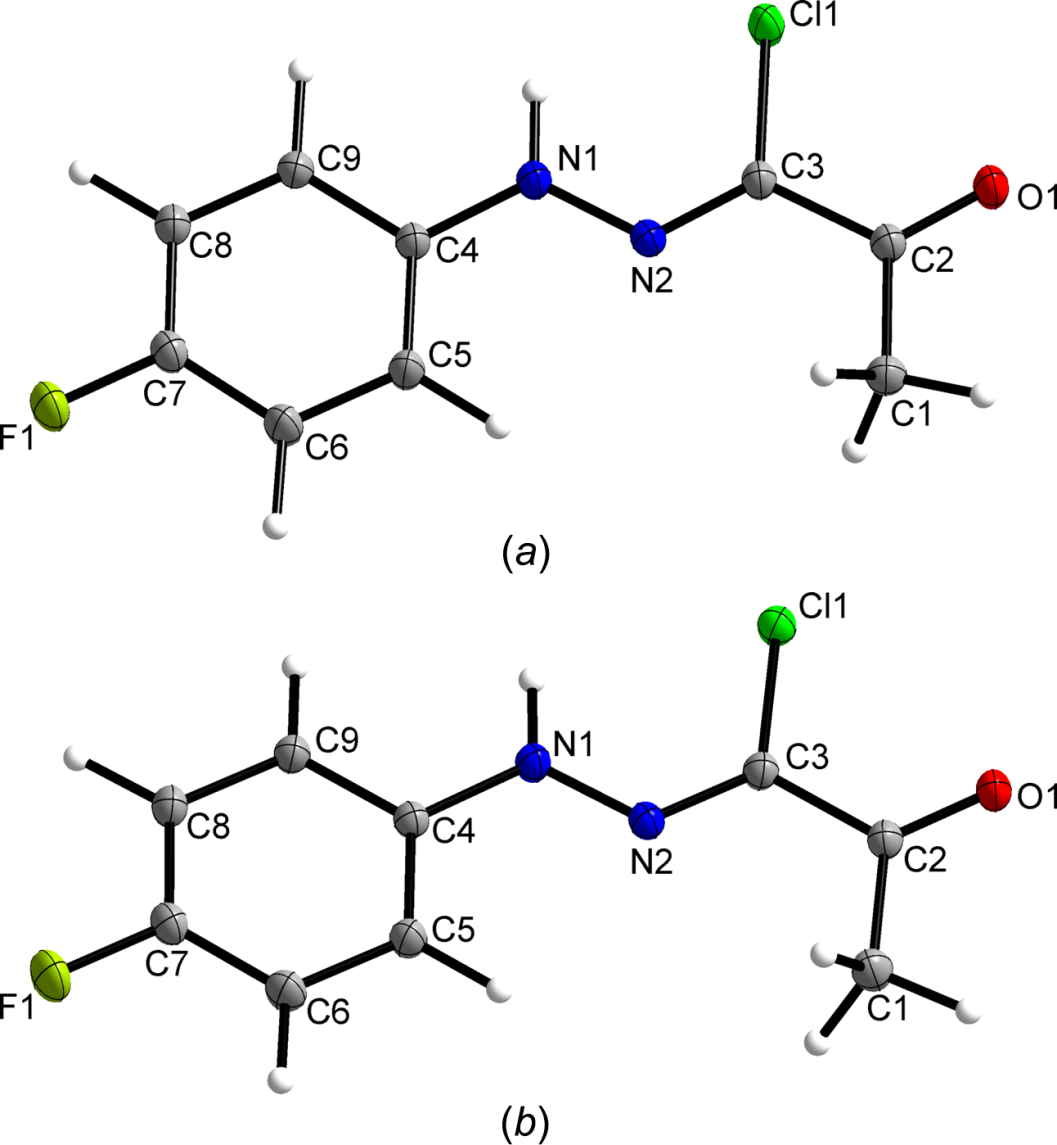
Displacement ellipsoid plots (50% probability) of (**1**) in (*a*) crystal form I and (*b*) crystal form II. H atoms are represented by small spheres of arbitrary radius.

**Figure 3:**
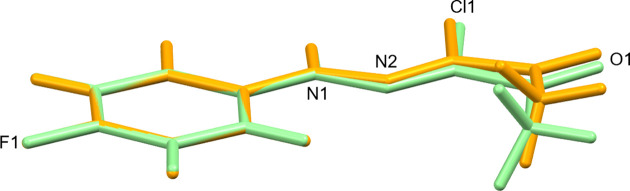
The mol­ecular structures of (**1**) in crystal forms I (orange) and II (light green) overlaid at the C atoms of the 4-fluorophenyl group. The r.m.s. deviation of the two mol­ecular structures from one another is 0.0925 Å.

Fig. 2 ▸ shows the mol­ecular structure of (**1**) in both crystal forms I and II, and Table 2 ▸ com­pares selected bond lengths and angles. The hydrazonoyl C=N double bond was found in the *Z* configuration in both forms, and the C=N and C=O moieties, as well as the C=N moiety and the 4-fluoro­phenyl group, adopt an *anti* conformation about the C2—C3 and N1—N2 formal single bonds, respectively. The same geometric arrangement of the hydrazonoyl chloride group was also exclusively encountered in the crystal structures of related *N*-phenyl-2-oxo­propane­hydrazonoyl chlorides (Asiri *et al.*, 2011*a* ▸, 2011*b* ▸, 2011*c* ▸, 2011*d* ▸; Abdel-Aziz *et al.*, 2012 ▸; Morjan *et al.*, 2013 ▸). In (**1**), the non-H-atom skeleton is essentially planar in both forms I and II, but the mol­ecule in form II adopts a conformation closer to exact *C*_s_ point-group symmetry (r.m.s. deviation = 0.0350 Å) than in form I (r.m.s. deviation = 0.0597 Å). The larger tilt of the hydrazonoyl group out of the plane of the arene group in form I com­pared to form II is also evident from the C5—C4—N1—N2 torsion angles (Table 2 ▸). The structure overlay plot shown in Fig. 3 ▸ illustrates the structural variation of the mol­ecular structure in both polymorphs. By com­parison, the C5—C4—N1—N2 torsion angle in the corresponding 4-meth­oxy­phenyl derivative (AWUXAS; Asiri *et al.*, 2011*a* ▸) is even larger than in form I at 10.8°. These deviations from planarity can be attributed to packing effects. In this context, it is worth noting that such minor conformational differences, as observed in forms I and II, do not lead to their classification as conformational polymorphs (Cruz-Cabeza & Bernstein, 2014 ▸).

**Figure 4:**
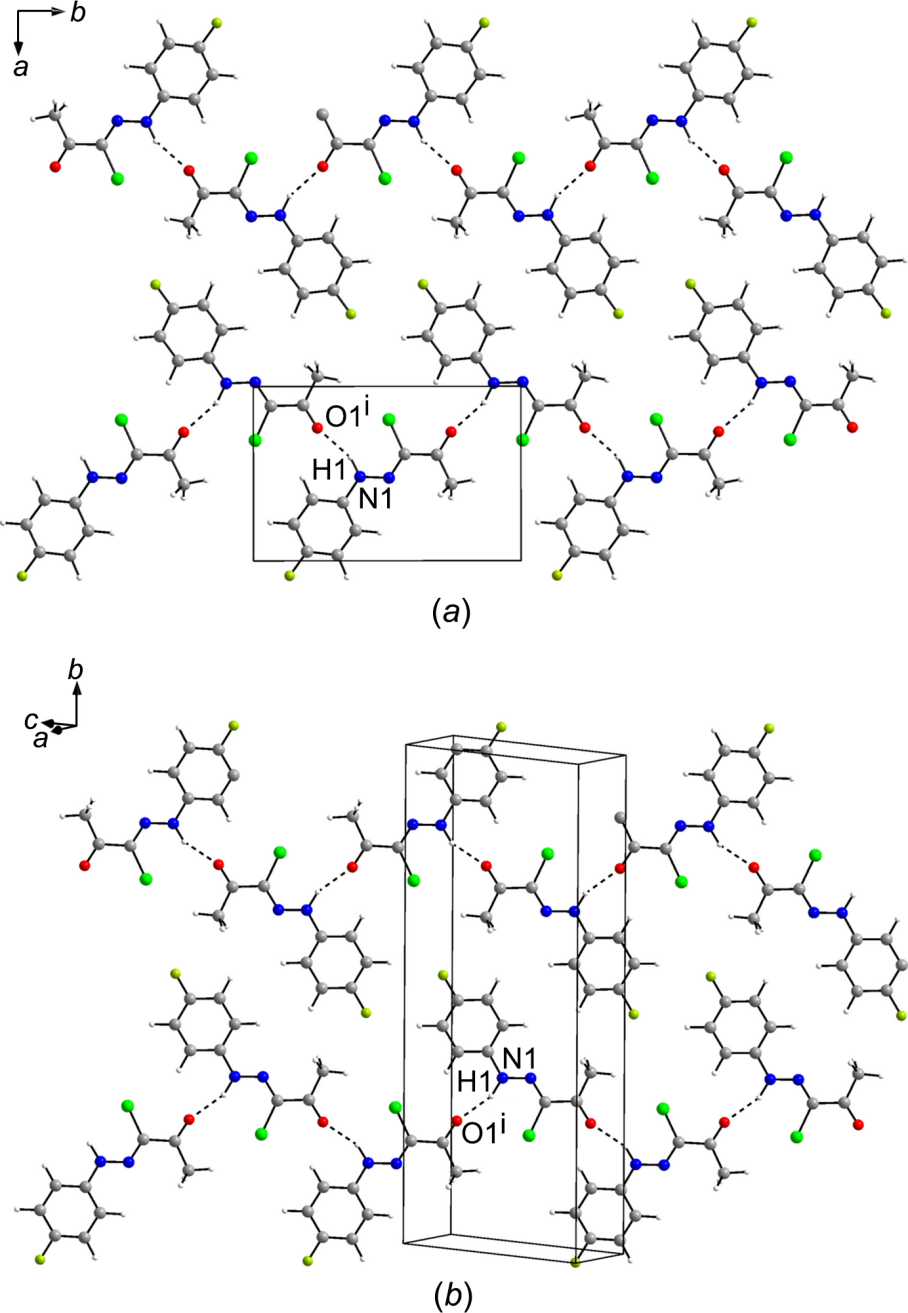
Hydrogen-bonded chains of (**1**) in (*a*) crystal form I (viewed along the *c*-axis direction) and (*b*) crystal form II [viewed towards the (11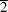) plane], shown in relation to the monoclinic unit cells. Hydrogen bonds are shown by dashed lines. Colour scheme: C grey, H white, Cl green, F lime, N blue and O red. [Symmetry codes: (i) −*x* + 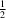, *y* − 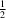, −*z* + 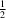 for part (*a*) and *x* + 1, −*y* + 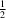, *z* + 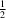 for part (*b*).]

**Figure 5:**
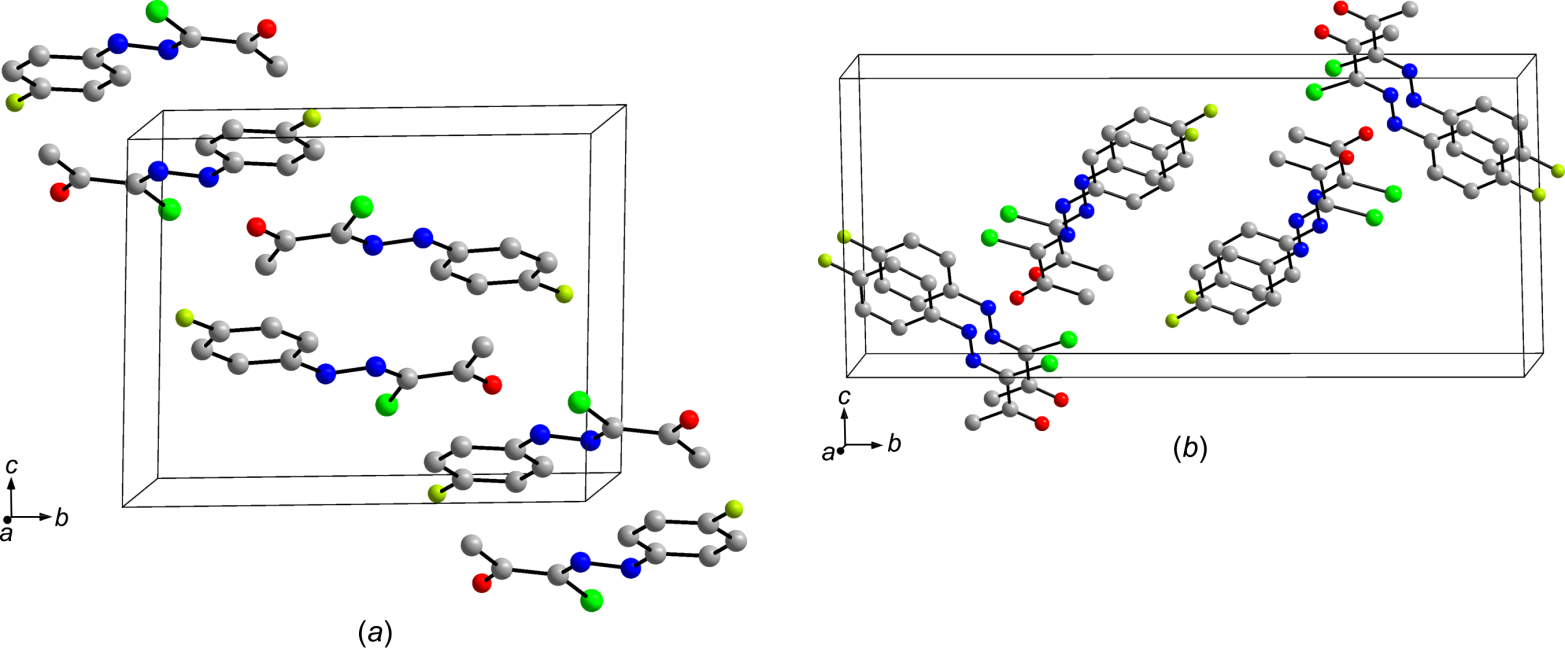
Packing diagrams of (**1**), showing the different spatial arrangement of two adjacent layers of the mol­ecules in (*a*) form I and (*b*) form II. H atoms have been omitted for clarity.

Polymorphs I and II of (**1**) both crystallize in the monoclinic system with one mol­ecule constituting the asymmetric unit (*Z*′ = 1). Hydrogen bonds of the N—H⋯O type are the prevailing inter­molecular inter­action in crystal forms I and II. The hydrazonoyl NH moiety forms a hy­dro­gen bond to the carbonyl O atom of an adjacent mol­ecule. As shown in Fig. 4 ▸, this results in zigzag chains with *C*(6) as the hy­dro­gen-bond motif descriptor (Bernstein *et al.*, 1995 ▸) in both forms I and II. The corresponding hy­dro­gen-bond parameters can be found in Tables 3 ▸ and 4 ▸. These fall within the expected ranges for strong hy­dro­gen bonds (Thakuria *et al.*, 2017 ▸). The hy­dro­gen-bonded chains so formed extend by 2_1_ screw symmetry in form I, as also encountered in the crystal structure of the corresponding unsubstituted phenyl derivative (XEBWIM; Abdel-Aziz *et al.*, 2012 ▸), and by glide symmetry in form II, as observed previously in the crystal structures of the 4-meth­oxy­phenyl (AWUXAS; Asiri *et al.*, 2011*a* ▸), 4-chloro­phenyl (AWUXEW; Asiri *et al.*, 2011*b* ▸) and 4-nitro­phenyl (AWUXIA; Asiri *et al.*, 2011*c* ▸) congeners. Although both polymorphs are characterized by parallel hy­dro­gen-bonded chains of mol­ecules in which the 4-fluoro­phenyl groups dovetail into one another to create layers, it is the spatial arrangement of the layers of mol­ecules so formed that distinguish the two polymorphs from one another. In form I, the mol­ecules in adjacent layers parallel to (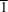
03) form anti­parallel dimers about inversion centres with a distance between the mean planes through the non-H atoms of 3.27 Å [Fig. 5 ▸(*a*)]. In contrast, in form II, the mol­ecules in adjacent layers parallel to (10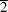) align in a parallel fashion with the mean planes through the mol­ecules separated by 3.40 Å [Fig. 5 ▸(*b*)]. Whereas the hy­dro­gen-bonded chains are vertically offset in form I, in form II the chains are arranged above each another (Fig. S2 in the supporting information). Since the difference between the mol­ecular conformations in both crystal forms is slight (*vide supra*), it is possible that crystal packing accounts for the different colours of polymorphs I and II (Nogueira *et al.*, 2020 ▸).

**Figure 6:**
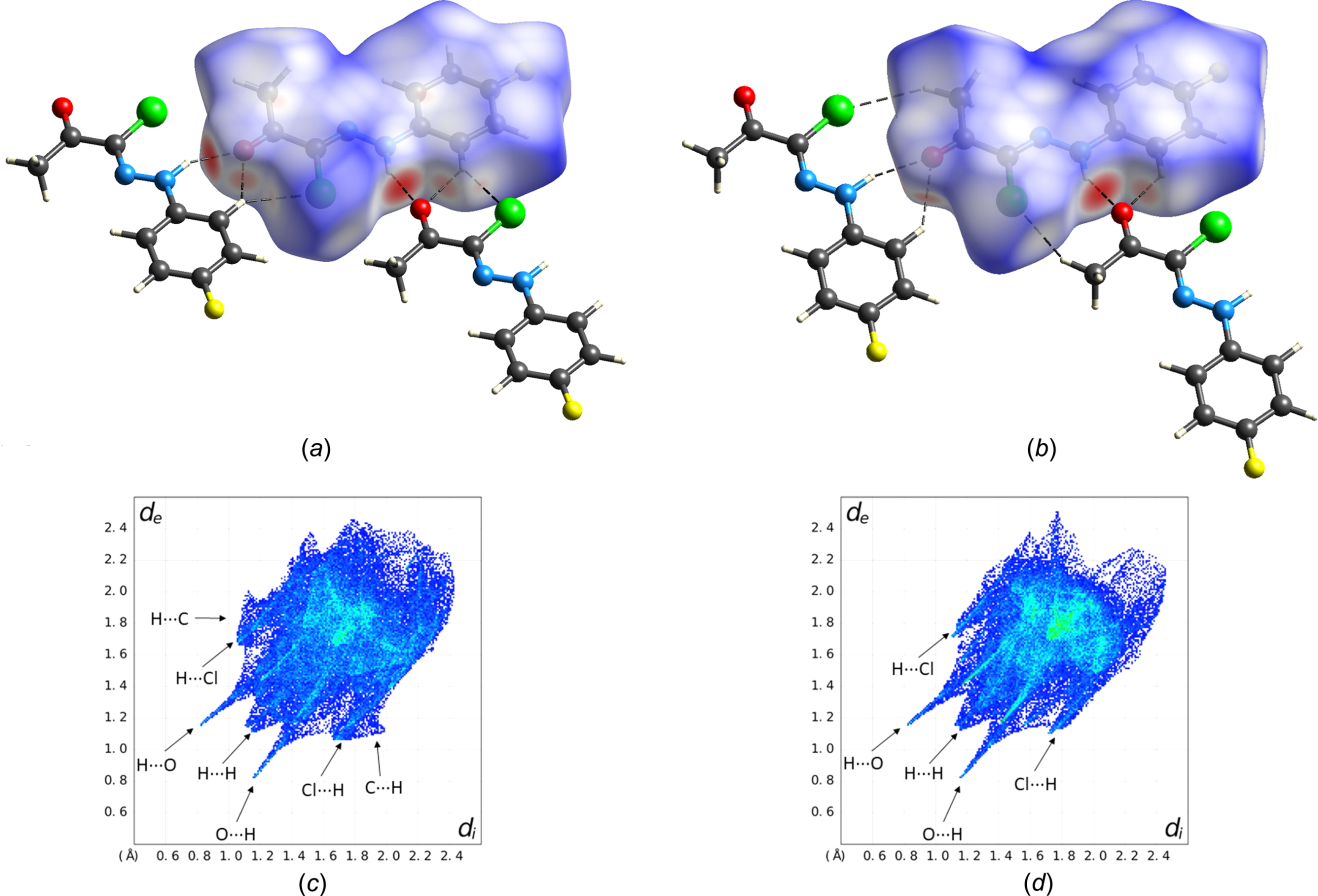
Hirshfeld surface mapped with *d*_norm_ for polymorph I (*a*) and II (*b*) of (**1**) and the corresponding *d*_e_*versus d*_i_ fingerprint plots (*c*)/(*d*). *d*_i_ and *d*_e_ are the distances from a point on the Hirshfeld surface to the nearest inter­nal and external atom, respectively. Colour scheme for the atoms: C grey, H white, Cl green, F yellow, N blue and O red.

To shed light on inter­molecular inter­actions in the crystal structures of both polymorphs by an objective identification of short contacts and in order to com­pare their supra­molecular solid-state structures in a more qu­anti­tative manner, Hirshfeld surface analysis was performed (Spackman & Jayatilaka, 2009 ▸). Hirshfeld surface plots mapped with the normalized contact distance (*d*_norm_) reveal a swapping of the shortest H⋯Cl distance from C9—H9⋯Cl1 in polymorph I [Fig. 6 ▸(*a*)] to C1—H1*A*⋯Cl1 in polymorph II [Fig. 6 ▸(*b*)]. Red and blue coloured areas in the *d*_norm_ plot indicate contacts respectively shorter and longer than the van der Waals contact distance of the nearest atoms to a point on the Hirshfeld surface. The large red areas correspond to the strong N—H⋯O hy­dro­gen bonds, while weak C—H⋯O and C—H⋯Cl hy­dro­gen bonds show up as minor red areas (*cf.* Tables 3 ▸ and 4 ▸). The corresponding fingerprint plots are depicted in Figs. 6 ▸(*c*) and 6(*d*). As expected, both have in common the large spikes resulting from the N—H⋯O hy­dro­gen bonds and the feature indicative of H⋯H contacts resulting from close packing. Wings that can be ascribed to C_meth­yl_—H⋯C_aromatic_ contacts are only present for form I, whereas small spikes from C—H⋯Cl contacts exist for both crystal forms. The central triangular feature on the diagonal of the fingerprint plot characteristic of C⋯C contacts from π–π stacking is more pronounced for form II, which is consistent with the observation that adjacent layers of mol­ecules are offset stacked in form I.

**Figure 7:**
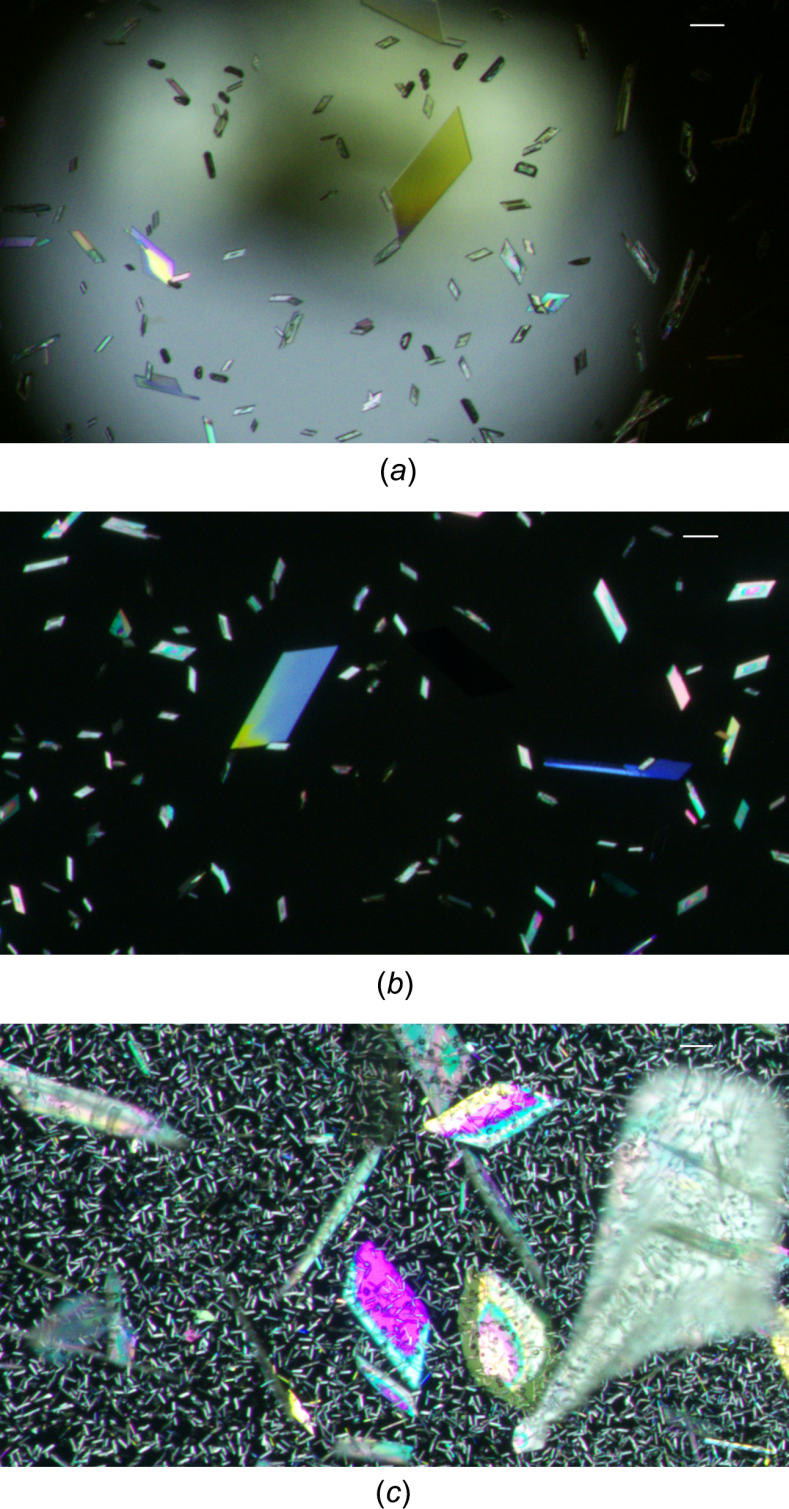
Crystals of (**1**) sublimed from (*a*)/(*b*) form I and (*c*) form II, showing similar morphology. Scale bars = 0.1 mm.

Notably, polymorphs I and II are virtually indistinguishable by their calculated densities (Table 1 ▸), their packing indices (Kitajgorodskij, 1973 ▸), *viz.* 72.6% for I and 72.9% for II, and their melting points as determined by hot-stage microscopy (*ca* 420 K) and DSC analysis (420.7 ± 0.3 K; see Fig. S3) (literature: 420–422 K, ethanol; Biere *et al.*, 1982 ▸). This suggests that the energy difference between the two polymorphs is small, which possibly accounts for their concomitant crystallization from acetone. Samples of both forms I and II began to sublime on the hot stage between about 373 K and the melting points. The crystals deposited by sublimation exhibited similar morphology and unit-cell parameters corresponding to those of form II (Figs. 7 ▸ and S4). As expected, both polymorphs are also nearly indistinguishable with respect to their IR spectra in the region 4000–400 cm^−1^ (see Fig. S5). The bands that can be assigned to the N—H and C=O stretching vibrations were observed at 3231 (form I) and 3234 (form II), and 1680 (form I) and 1678 cm^−1^ (form II), respectively. Since mid-IR spectroscopy mainly probes vibrations associated with functional groups, it cannot readily discriminate between polymorphs with similar mol­ecular structures and similar hy­dro­gen-bonding patterns as observed here (Suresh *et al.*, 2019 ▸).

## Conclusions

We have discovered concomitant colour polymorphism of com­pound (**I**) by serendipity. X-ray crystallography revealed that the two colour polymorphs have in common a nearly planar mol­ecular conformation of the non-H-atom skeleton and the inter­molecular one-periodic hy­dro­gen-bonding pat­tern. In contrast, the spatial arrangement of the hy­dro­gen-bonded chains is distinctly different in both polymorphs, albeit with virtually similar packing efficiency, as revealed by the calculated densities and packing indices. Polymorph I is characterized by offset stacking of the mol­ecules with an anti­parallel alignment between the closest mol­ecules, whereas the mol­ecules in polymorph II stack in a parallel fashion. The results suggest that the colour difference between the two polymorphs may result from the different crystal packing rather than different mol­ecular conformations in the solid state.

## Supplementary Material

Crystal structure: contains datablock(s) I, II, global. DOI: 10.1107/S2053229625006618/oc3027sup1.cif

Structure factors: contains datablock(s) I. DOI: 10.1107/S2053229625006618/oc3027Isup2.hkl

Structure factors: contains datablock(s) II. DOI: 10.1107/S2053229625006618/oc3027IIsup3.hkl

Supporting information file. DOI: 10.1107/S2053229625006618/oc3027Isup4.cdx

Supporting information file. DOI: 10.1107/S2053229625006618/oc3027IIsup5.cdx

Supporting information file. DOI: 10.1107/S2053229625006618/oc3027Isup6.cml

Additional figures and spectra. DOI: 10.1107/S2053229625006618/oc3027sup7.pdf

CCDC references: 2475376, 2475377

## Figures and Tables

**Table 1:** Experimental details For both determinations: C_9_H_8_ClFN_2_O, *M*_r_ = 214.63, *Z* = 4. Experiments were carried out with Mo *K*α radiation using a Bruker D8 VENTURE diffractometer. The absorption correction was Gaussian (*SADABS*; Bruker, 2016 ▸). All H-atom parameters were refined.

	(I)	(II)
Crystal data		
Crystal system, space group	Monoclinic, *P*2_1_/*n*	Monoclinic, *P*2_1_/*c*
Temperature (K)	100	101
*a*, *b*, *c* (Å)	8.3284 (10), 12.0862 (15), 9.8144 (14)	3.8945 (3), 23.3985 (15), 10.2692 (7)
β (°)	108.762 (7)	94.235 (3)
*V* (Å^3^)	935.4 (2)	933.23 (11)
*D_x_* (Mg m^−3^)	1.524	1.528
μ (mm^−1^)	0.39	0.39
Crystal shape	Block	Prism
Colour	Pale orange	Pale yellow
Crystal size (mm)	0.33 × 0.23 × 0.1	0.33 × 0.13 × 0.08

Data collection		
*T*_min_, *T*_max_	0.919, 0.974	0.938, 0.977
No. of measured, independent and observed [*I* ≥ 2σ(*I*)] reflections	260021, 2884, 2561	40492, 2872, 2641
*R*_int_	0.058	0.050
(sin θ/λ)_max_ (Å^−1^)	0.718	0.715

Refinement		
*R*[*F*^2^ > 2σ(*F*^2^)], *wR*(*F*^2^), *S*	0.024, 0.075, 1.19	0.030, 0.066, 1.05
No. of reflections	2884	2872
No. of parameters	184	159
Δρ_max_, Δρ_min_ (e Å^−3^)	0.29, −0.21	0.24, −0.26

Computer programs: *APEX5* (Bruker, 2022 ▸), *SAINT* (Bruker, 2019 ▸), *SHELXT* (Sheldrick, 2015*a* ▸), *olex2.refine* (Bourhis *et al.*, 2015 ▸), *DIAMOND* (Brandenburg, 2018 ▸), *Mercury* (Macrae *et al.*, 2020 ▸) and *publCIF* (Westrip, 2010 ▸).

**Table 2:** Selected bond lengths and angles (Å, °) for polymorphs I and II

	Form I	Form II
C1—C2	1.5018 (11)	1.5044 (16)
C2—C3	1.4770 (12)	1.4724 (16)
C2—O1	1.2194 (10)	1.2192 (13)
C3—Cl1	1.7366 (9)	1.7342 (11)
C3—N2	1.2856 (10)	1.2908 (14)
C4—N1	1.4007 (10)	1.4055 (14)
N1—N2	1.3213 (9)	1.3117 (13)
		
C3—C2—C1	118.12 (7)	117.57 (10)
O1—C2—C1	122.66 (7)	122.46 (11)
Cl1—C3—C2	116.39 (6)	117.17 (8)
N2—C3—C2	121.30 (7)	119.60 (10)
N2—N1—C4	120.59 (7)	119.48 (9)
N1—N2—C3	120.28 (7)	121.70 (10)
		
C1—C2—C3—Cl1	178.23 (7)	−176.28 (9)
C1—C2—C3—N2	−2.23 (9)	3.02 (13)
C2—C3—N2—N1	−179.70 (8)	179.93 (10)
C3—N2—N1—C4	−178.69 (8)	−178.87 (11)
C5—C4—N1—N2	−5.56 (10)	−1.46 (13)

**Table 3:** Hydrogen-bond geometry (Å, °) for form I[Chem scheme1]

*D*—H⋯*A*	*D*—H	H⋯*A*	*D*⋯*A*	*D*—H⋯*A*
N1—H1⋯O1^i^	0.973 (14)	2.013 (14)	2.9284 (10)	155.9 (11)
C1—H1*a*⋯Cl1^ii^	1.057 (18)	2.922 (18)	3.7917 (11)	139.9 (12)
C1—H1*c*⋯N1^iii^	1.042 (15)	2.805 (15)	3.5597 (12)	129.5 (10)
C1—H1*c*⋯O1^iv^	1.042 (15)	2.695 (15)	3.5188 (12)	135.9 (11)
C8—H8⋯F1^v^	1.071 (14)	2.653 (14)	3.6161 (11)	149.3 (10)
C9—H9⋯Cl1^i^	1.067 (14)	2.757 (15)	3.7178 (10)	149.7 (11)
C9—H9⋯O1^i^	1.067 (14)	2.355 (14)	3.2404 (11)	139.3 (11)

Symmetry codes: (i) 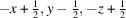; (ii) 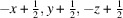; (iii) 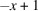, 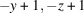; (iv) 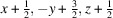; (v) 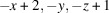.

**Table 4:** Hydrogen-bond geometry (Å, °) for form II[Chem scheme1]

*D*—H⋯*A*	*D*—H	H⋯*A*	*D*⋯*A*	*D*—H⋯*A*
N1—H1⋯O1^i^	0.993 (16)	1.992 (16)	2.9196 (13)	154.5 (13)
C1—H1*a*⋯Cl1^ii^	1.07 (2)	2.83 (2)	3.7787 (13)	148.2 (15)
C1—H1*b*⋯N2^iii^	1.042 (19)	2.744 (19)	3.5452 (17)	133.7 (13)
C1—H1*c*⋯F1^iv^	1.05 (2)	2.63 (2)	3.6063 (15)	153.9 (15)
C8—H8⋯F1^v^	1.079 (16)	2.576 (17)	3.6171 (14)	161.8 (12)
C9—H9⋯O1^i^	1.066 (17)	2.364 (17)	3.2335 (14)	137.8 (13)

Symmetry codes: (i) 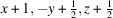; (ii) 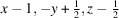; (iii) 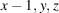; (iv) 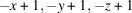; (v) 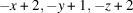.
