# Supplementary material for: Concomitant colour polymorphs of (Z)-N-(4-fluoro­phen­yl)-2-oxo­propane­hydrazonoyl chloride
Source: Acta Crystallogr C Struct Chem. 2025 Jul 30;81(Pt 8):481–7. doi: 10.1107/S2053229625006618 (PMC12322930; doi:10.1107/S2053229625006618)
Supplement: Supplementary file 7 [file c-81-00481-sup7.pdf]

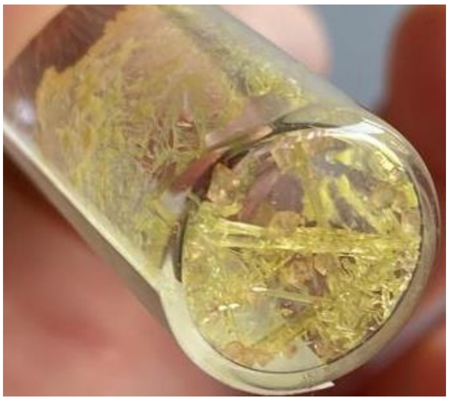

a)

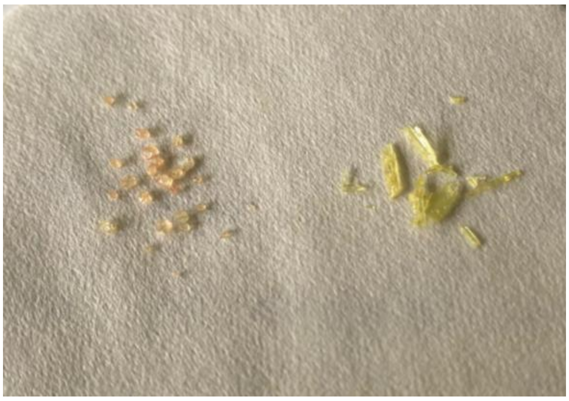

b)

Figure S1. a) The two concomitantly crystallized colour polymorphs of the title compound in the crystallization vessel. b) Manually separated pale orange crystals of polymorph I (left) and pale yellow crystals of polymorph II (right).

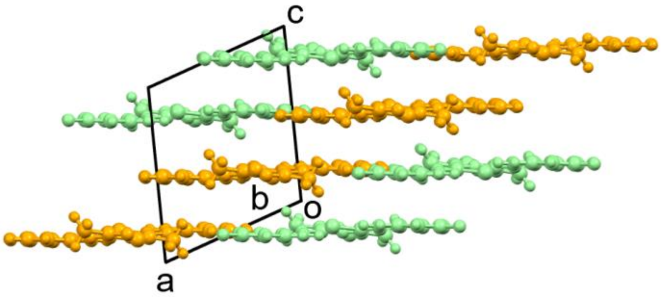

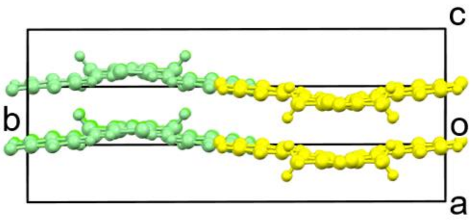

Figure S2. View along the N–H···O hydrogen-bonded chains of form I (left) and form II (right) showing the stacking of the chains in the respective unit cells. Chains with the N–H groups pointing to the viewer are shown in light green, whereas those pointing away from the viewer are shown in light orange (form I) or yellow (form II).

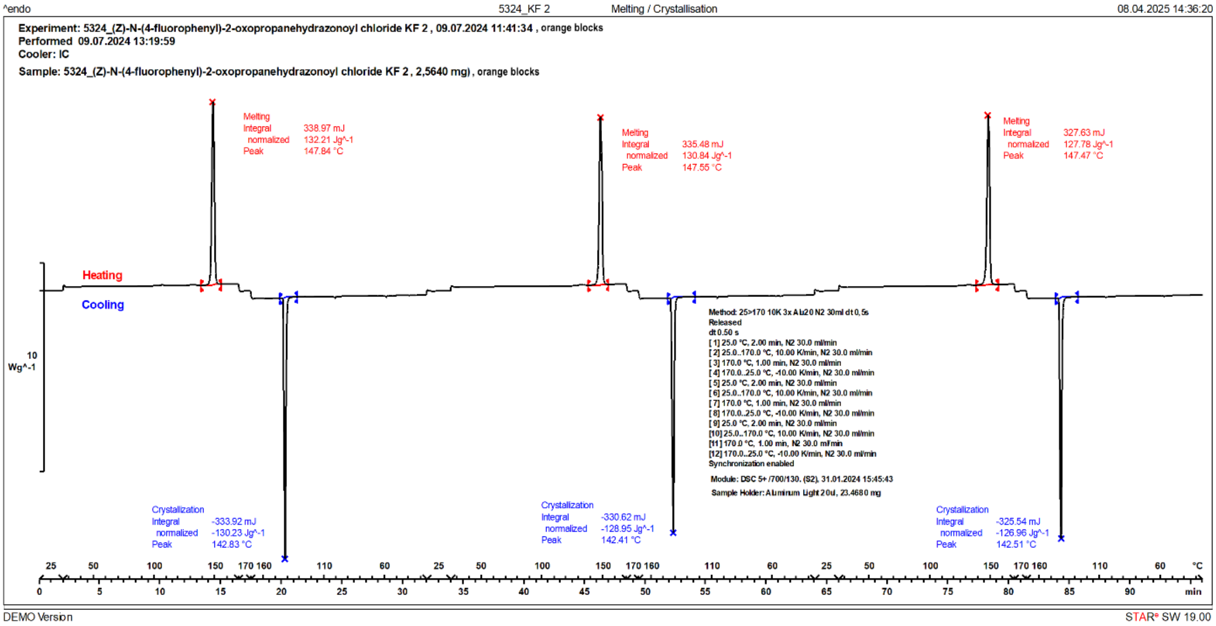

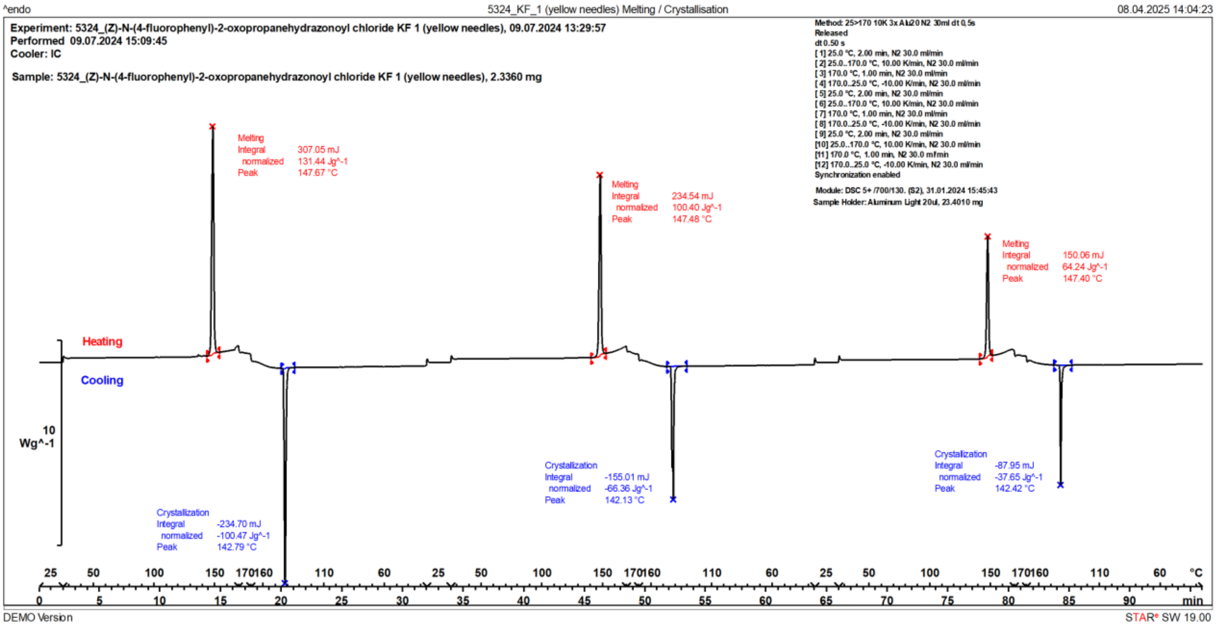

Figure S3. DSC analyses of form I (top) and form II (bottom).

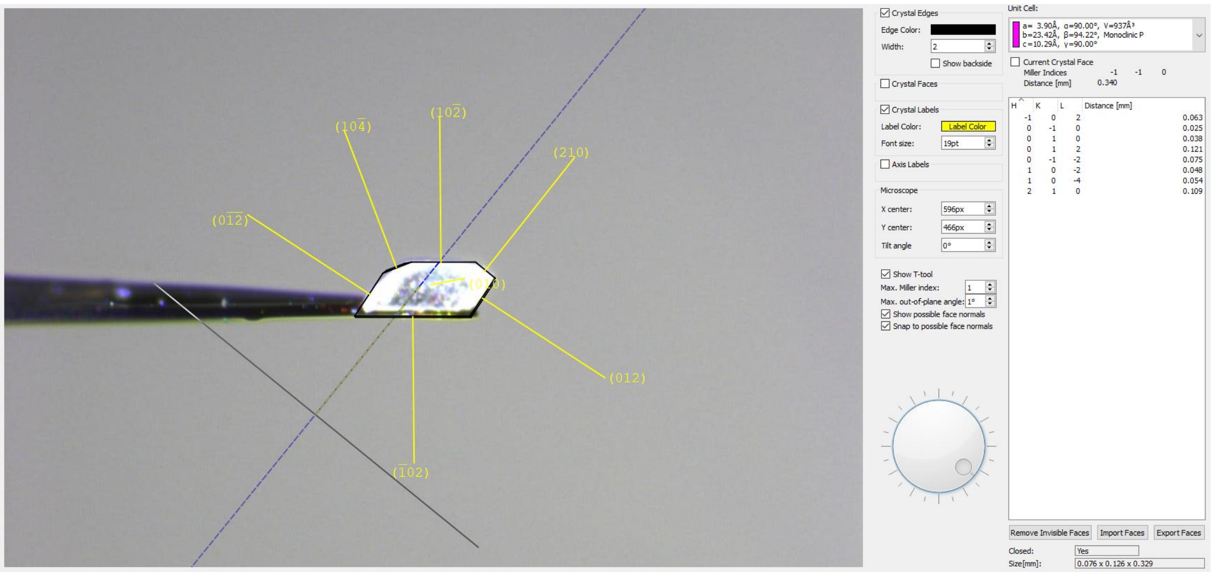

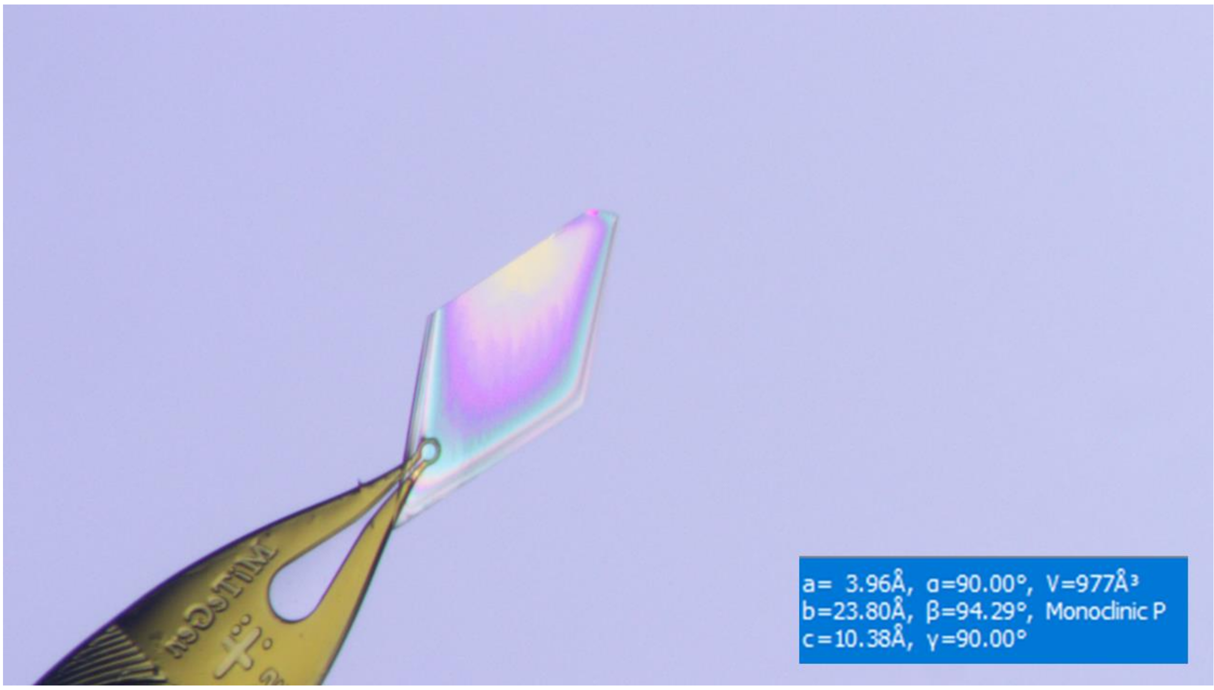

Figure S4. Crystal of form II used for data collection (top) and crystal of sublimed form I exhibiting the unit cell parameters of form II (bottom).

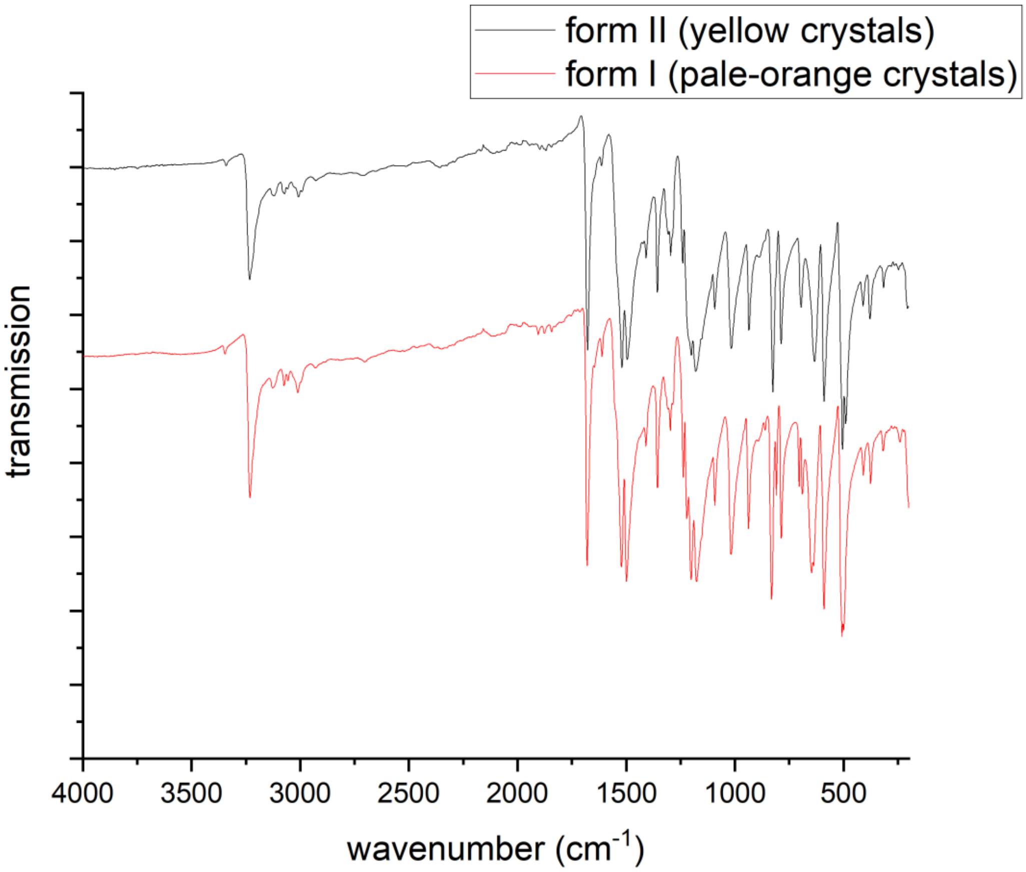

Figure S5. Infrared spectra of form I (red) and form II (black).
